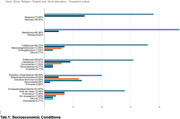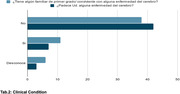# Validation of a questionnaire to measure the attitude of the Peruvian population regarding brain donation for the purpose of investigation

**DOI:** 10.1002/alz.093356

**Published:** 2025-01-03

**Authors:** Lucia Bulgarelli, Ernesto Benavidez‐Carbajal, Fiorella Canevaro‐Sesarego, Ethel Rodriguez‐Lopez, Claudia Cruzalegui‐Bazán, Claudia Mejia‐Rojas, Marzhio Campos, Eddy De La Cruz, Koni Mejia‐Rojas

**Affiliations:** ^1^ Università degli studi di Padova, Padova Italy; ^2^ EDMECON Continuing Medical Education, Lima Peru; ^3^ National University of San Marcos, Lima Peru; ^4^ National Rehabilitation Institute “Dra. Adriana Rebaza Flores” FRIENDSHIP PERU ‐ JAPAN, Lima Peru; ^5^ Neurogenetics Research Center, Instituto Nacional de Ciencias Neurológicas, Lima Peru; ^6^ Daniel Alcides Carrion National Hospital, Callao Peru

## Abstract

**Background:**

Research into human brain tissue is important for medical scientific advancement. Brain Banks worldwide allow the receipt of brains for such purposes through voluntary donation. Attitude towards organ donation can be influenced by cultural, ethical and social factors. The lack of a validated questionnaire to evaluate it in the Peruvian population highlights the need for this study in the context of the creation of the First Brain Bank in Peru.

**Method:**

An observational, descriptive cross‐sectional study was carried out.

The questionnaire was applied to 52 Peruvians over 18 years of age, selected by convenience in different areas of Peru, who agreed to participate voluntarily.

The questionnaire developed took as reference the “Health Street” questionnaire, created by the University of Florida. Then adapted taking into consideration the Peruvian context, according to the comments of experts on the subject. It resulted in 43 items with responses via Likert scale, 1 dichotomous, 10 polytomous and 3 open.

The questionnaire was applied to the participants through an in‐person and/or virtual interview. Likewise, after signing the informed consent, in some cases the administration was guided by the researcher, and in others it was self‐administered.

Finally, the internal consistency of the instrument was evaluated using Cronbach’s Alpha coefficient.

**Result:**

The questionnaire was applied to 52 Peruvians with different socioeconomic and clinical conditions (tab.1 and tab.2), recollected from different parts of the country and whose average age was 38.61 years.

The Cronbach’s alpha coefficient calculation was 0.97.

**Conclusion:**

The evaluation of the questionnaire by the experts was adequate: there were 5 experts, as recommended in the literature.

This questionnaire demonstrated good reliability in relation to the questions (Cronbach’s alpha was 0.97) so there is a good acceptability of the instrument.